# Choosing an epidemiological model structure for the economic evaluation of non-communicable disease public health interventions

**DOI:** 10.1186/s12963-016-0085-1

**Published:** 2016-05-04

**Authors:** Adam D. M. Briggs, Jane Wolstenholme, Tony Blakely, Peter Scarborough

**Affiliations:** BHF Centre on Population Approaches for Non-Communicable Disease Prevention (BHF CPNP), Nuffield Department of Population Health, University of Oxford, Old Road Campus, Headington, Oxford, OX3 7LF UK; Health Economics Research Centre (HERC), Nuffield Department of Population Health, University of Oxford, Oxford, UK; Health Inequalities Research Programme (HIRP), Department of Public Health, University of Otago, Wellington, New Zealand

**Keywords:** Modeling, Cost-effectiveness, Non-communicable disease, Economics, Public health

## Abstract

Non-communicable diseases are the leading global causes of mortality and morbidity. Growing pressures on health services and on social care have led to increasing calls for a greater emphasis to be placed on prevention. In order for decisionmakers to make informed judgements about how to best spend finite public health resources, they must be able to quantify the anticipated costs, benefits, and opportunity costs of each prevention option available. This review presents a taxonomy of epidemiological model structures and applies it to the economic evaluation of public health interventions for non-communicable diseases. Through a novel discussion of the pros and cons of model structures and examples of their application to public health interventions, it suggests that individual-level models may be better than population-level models for estimating the effects of population heterogeneity. Furthermore, model structures allowing for interactions between populations, their environment, and time are often better suited to complex multifaceted interventions. Other influences on the choice of model structure include time and available resources, and the availability and relevance of previously developed models. This review will help guide modelers in the emerging field of public health economic modeling of non-communicable diseases.

## Introduction

Non-communicable diseases (NCDs) are the leading causes of mortality and morbidity globally, with much of this disease burden being preventable [1–3]. Health services around the world are experiencing ever-increasing demand that may be alleviated through placing a greater emphasis on NCD prevention. For example, in 2014 the National Health Service (NHS) in England announced the need for a “radical upgrade in prevention and public health” [4] and in 2005, the UK National Institute for Health and Care Excellence (NICE) started a new initiative to produce guidance on public health interventions, including cost-effectiveness analyses [5]. As of January 2016, 59 public health guidelines had been published [6].

In order for decisionmakers to make informed choices about how best to spend finite public health resources, they need to be able to quantify the anticipated impact of an intervention, its cost, the associated opportunity cost, and the possible effect on inequalities. Decisionmakers then need to be able to directly compare two or more public health policies. This is difficult using current public health economic models due to differences in the underlying modeling method used (from here on referred to as the model structure), time horizon, epidemiological parameters, and outcome and costing measures. Therefore standardized processes are required for assessing and modeling the cost, health impact, and possibly cost-effectiveness of public health interventions affecting NCDs. NICE and the NICE Decision Support Unit have set out guidelines for public health economic modeling, but these are broadly based on guidelines for health technology assessments (HTAs) [7–9]. There are further difficulties faced by NICE and public health modelers: firstly, the level of evidence required by health technology assessments (randomized controlled trials) may not be possible to achieve and using Bayesian methods may be a robust and more appropriate approach to take [10, 11]; secondly, the topic addressed by the public health guidance is commonly dictated by the needs of the decisionmaker rather than on the availability of appropriate data. As a result, NICE guidance often fails to sufficiently address some of the specific challenges of modeling economic evaluations of public health interventions [12–19]. These are:

- long-term health impacts (e.g., the effects on cardiovascular disease burden and health expenditure of increasing the price of unhealthy foods may manifest and persist for many years after the intervention is introduced);
- wider societal costs and consequences (e.g., transport policies to improve physical activity have social costs and outcomes beyond health; and different social costs and outcomes are relevant to different modeled scenarios, for example, influencing alcohol use has an important effect on crime whereas reducing stroke incidence has a more important effect on social care);
- impact on inequalities (e.g., more deprived population groups may respond differently to those who are less deprived following a price increase on unhealthy food);
- multicomponent interventions (e.g., a policy aimed at increasing physical activity may include both additional bicycle lanes and subsidized gym membership); and
- interactions within complex non-health sector systems (e.g., if simulating the effect of upgrading home insulation to reduce winter deaths, it may be important to consider interactions with the housing sector, energy sector, and social care).

Examples of models are used throughout this review to highlight how different model structures cope with one or more of these challenges.

**Fig. 1:**
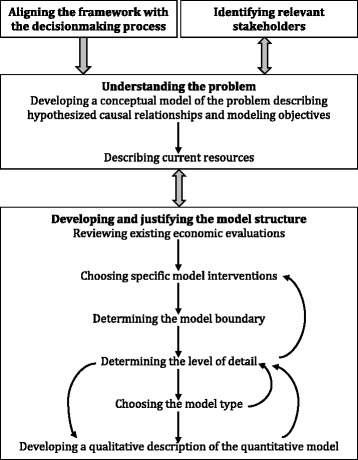
Squires’ conceptual modeling framework for public health economic modeling. Reproduction of figure 7.3 in Squires, 2014 (with permission from the author) [22]. Legend: The figure describes how to develop a public health economic model. Model development should be an iterative process as new stakeholders and data are identified, represented by the double-headed arrows

The International Society for Pharmacoeconomics and Outcomes Research-Society for Medical Decision Making (ISPOR-SMDM) guidelines for modeling research present a key set of papers and guidelines on best practice in health care decision modeling [20]. These guidelines also include how to approach model transparency and evaluation, but these topics are not covered in our review [21]. The ISPOR-SMDM guidelines, as with those produced by NICE, are primarily focused on health technology appraisals and do not address the specific challenges of public health interventions. Squires outlines these problems in more detail and presents a framework for developing public health economic models (Fig. 1) [22].

Squires emphasizes that when using this framework, it is important to adopt an iterative approach (represented by the double-headed arrows and the arrows feeding back) as in practice, the understanding of the problem to be modeled will develop as the model is built. Decisions regarding model inputs, outputs, scope, and structure are influenced by several processes and considerations. These include understanding exactly what should be modeled, views of stakeholders, data availability, and what other models are currently available (where appropriate, the development of new models should be avoided if existing models can be used or adapted) [22].

## A taxonomy of epidemiological modeling structures for the economic evaluation of public health interventions

Authors have previously developed taxonomies to help modelers to decide on the most appropriate health economic model structure, however, these are primarily aimed at HTAs [19, 23, 24] and as discussed in the introduction, are difficult to apply to public health interventions for NCDs. In order to make Brennan’s taxonomy more relevant to public health economic modeling, Squires adapted it to include agent-based simulation [22, 24]. In this paper we use Squires’ adapted version of Brennan et al.’s taxonomy as a framework for discussing public health economic modeling of NCDs. We add comparative risk assessment (CRA) models, which can simultaneously model multiple disease processes and risk factors without large increases in model complexity, to the taxonomy (Table 1). In the descriptions of model structures, we also include a discussion of how microsimulation techniques and multistate life tables can be a useful adjunct to some of the model structures in Table 1. We are aware that other model combination models are possible, for example using the outputs of a decision tree to parameterise a Markov model [25]; however, these are not individual model structures in themselves and all such combinations are therefore not included in this review.

**Table 1:** Revised version of Brennan’s taxonomy of model structures [22, 24]

			A	B	C	D
			Cohort/aggregate-level/counts		Individual-level	
			Expected value, continuous state, deterministic	Markovian, discrete state, stochastic	Markovian, discrete state	Non-Markovian, discrete state
1	No interaction	Untimed	Decision tree rollback or comparative risk assessment	Simulation decision tree or comparative risk assessment	Individual sampling model: Simulated patient-level decision tree or comparative risk assessment	
2		Timed	Markov model (deterministic)	Simulation Markov model	Individual sampling model: Simulated patient-level Markov model	
3	Interaction between entity and environment	Discrete time	System dynamics (finite difference equations)	Discrete time Markov chain model	Discrete-time individual event history model	Discrete-time discrete event simulation
4		Continuous time	Systems dynamics (ordinary differential equations)	Continuous time Markov chain model	Continuous time individual event history model	Continuous-time discrete event simulation
5	Interaction between heterogeneous entities/spatial aspects important		x	x	x	Agent-based simulation

**Table 2:** Summary table of epidemiological modeling structures for the economic evaluation of non-communicable disease public health interventions

Corresponding section of review and table 1	Modeling method	Advantages	Disadvantages	Public health examples
Section: Decision trees	Decision tree	Can be easy to construct.	No explicit time component.	Comparing exercise referral schemes with usual care to increase physical activity [29].
		Relatively easy to interpret.	Exponentially more complex with additional disease states.	
Table 1: A1, B1, C1, D1		Can be adapted for cohorts and individuals.	No looping/recurring.	
			Poorly suited to complex scenarios.	
Section: Comparative riskassessment	Comparative risk assessment	Can model multiple diseases and risk factors simultaneously.	More complex to build than decision trees.	Return on investment of workplace interventions to improve physical activity [32].
		Can be used for individuals or cohorts.	No explicit time component.	
			No looping/recurring.	
Table 1: A1, B1, C1, D1			Unable to model interactions between individuals, populations, or their environment.	
Section: Markov models without interaction	Markov models without interaction	Relatively straightforward to construct and to communicate.	The Markovian assumption-individuals have no memory of (are independent of) previous disease states.	Investigating the cost effectiveness of different smoking cessation strategies using the Benefits of Smoking Cessation on Outcomes (BENESCO) model [33–35].
		Can model populations or individuals.		
Table 1: A2, B2, C2, D2		Has time component.	Can only exist in one disease state.	
		Allows looping/recurring.	Exponential increase in complexity with increasing numbers of disease states.	
Section: System dynamics models	System dynamics models	Allows for interactions between populations and the environment.	Models populations rather than individuals.	Modeling the effects of policies aimed at increasing bicycle commuting rather than travelling by car [63].
Table 1: A3, A4		Allows for feedback and recurring.		
Section: Markov chain models and individual-level Markov models with interaction	Markov chain models and Markov individual event history models	Can model individuals or populations.	Markovian assumption still exists (although its impact can be reduced-see main text).	A CDC model evaluating the cost-effectiveness of different diabetes prevention strategies [58, 59].
Table 1: B3, B4, C3, C4		Allows for interaction between populations or individuals within the model.	Becomes rapidly more complex with added disease states.	
Section: Discrete event simulation	Discrete event simulation	Allows for interaction between individuals and between individuals, populations, and their environment, governed by system rules.	Model structure can be difficult to communicate and interpret.	Evaluating the cost-effectiveness of screening programs [67].
Table 1: D3, D4			Computationally challenging both in terms of designing the model and running it.	
		Allows for modeling of complex scenarios.		
Section: Agent-based simulation	Agent-based simulation	Allow for interactions within and between individuals, populations, and the environment, governed by rules applied to individuals.	More complex than discrete event simulation.	The Archimedes model for modeling the outcomes of changing health care systems, such as investigating diabetes care [70].
Table 1: D5			Requires large computational power.	
		Allows for individuals to learn.	Difficult to communicate and interpret model structure.	
		Allows modeling of complicated systems.		
Table 1: adjunct to A1, B1, C1, D1, A2, B2, C2, D2	Multistate life tables	Can be used with comparative risk assessment and decision tree models to add a time component.	Assumes diseases are independent of each other.	The Australian Assessing Cost Effectiveness in Prevention (ACE Prevention) project [74, 76].
		Can be combined with Markov models to increase the numbers of possible disease states without exponentially increasing model complexity.	Model limited by underlying model structure, for example, if combined with a Markov model, the Markovian assumption remains.	
Table 1: adjunct to C1, C2, C3, C4, D1, D2	Microsimulation	Can be combined with decision tree, comparative risk assessment, and Markov models to make it easier to model heterogeneous populations or multiple disease states.	Data requirements and simulations can become computationally challenging with complex models.	The NICE obesity health economic model used by Trueman et al. to estimate the cost-effectiveness of primary care weight management programs [83].
			Model limited by underlying model structure, for example, if combined with a Markov model, the Markovian assumption remains.	

In Table 1, columns A to D divide models into population- or individual-level, and separate model structures by how they deal with random events, expected values, and heterogeneous populations. Broadly speaking, by using population-level model structures, population heterogeneity (differences between population subgroups; for example, in terms of age, gender, or risk factors) can be simulated by rerunning the model for different cohorts, whereas individual-level model structures use multiple samples of different types of people (see section: *Use of microsimulation with individual-level decision tree, comparative risk assessment, and Markov models*). The ability to incorporate randomness allows for Monte Carlo simulations to estimate stochastic uncertainty (describing the uncertainty in individual level models resulting from two individuals being in the same situation but, by chance, having different outcomes), and parametric uncertainty (in either population- or individual-level models and describing the uncertainty in the estimates of model parameters) [26]. Rows 1–4 categorize model structures by whether they allow for interactions to occur between entities within the model and between entities and the environment, and how the model deals with time (untimed means the models do not explicitly include a temporal component). Row 5 describes agent-based simulation modeling, which allows for multiple interactions governed by rules affecting individuals within the model, rather than affecting the system, as is the case in rows 3 and 4. Table 2 summarizes the main advantages and disadvantages of the epidemiological modeling structures discussed in this review.

This review presents an analysis of the pros and cons of epidemiological modeling structures giving examples of their application to the economic evaluation of public health interventions for NCDs to guide modelers in the field of public health economic modeling.

## Description of model structures

### Rows 1 and 2 – no interaction

#### Decision trees (row 1, columns A, B, C, and D)

Decision trees simulate possible decisions and outcomes using branches to represent each potential event. Branch points are usually described as nodes and can represent a decision (i.e., whether or not a public health education campaign takes place) or a chance event (i.e., development of disease). The options at each chance node are assumed to be mutually exclusive and the probability of each option occurring needs to sum to one. Each branch eventually ends with a terminal node against which the associated morbidity and costs of that patient journey can be attached [27]. In order to calculate the cost-effectiveness of an intervention, the costs and morbidity of each patient journey are multiplied by the probability of that journey occurring, and then summed for each intervention and compared.

Decision trees are one of the simpler model structures available for public health economic modeling, and as such they are transparent as well as relatively straightforward to construct and to analyze. Decision trees can operate at the cohort level or at the individual level but do not have any explicit time component, do not allow for looping (recurring) of events, and do not accommodate interactions between individuals or populations [24]. This makes it particularly challenging to model long term chronic conditions or relapses in disease without becoming overly complicated [27].

Although often used by health economists, decision trees are less commonly used in public health economic models as they are particularly constrained by their lack of a temporal component, which other model structures can handle more efficiently [22, 28]. Trueman and Anokye used a decision tree to model exercise referral schemes (ERSs) for promoting physical activity in the UK [29]. Alongside a cost-utility analysis, Trueman and Anokye estimated the effects of ERSs on a variety of health outcomes and work absenteeism using a cost-consequence analysis. A cost-consequence analysis (CCA) is a useful way to illustrate the potential impact of public health interventions on wider society that may be relevant to public health decision makers but are not amenable to being quantified through standard measures of quality of life (such as those used to calculate Quality Adjusted Life Years [QALYs]). However, CCAs do not allow for comparisons across disease areas where some diseases include wider societal costs and benefits, and other diseases do not.

#### Comparative risk assessment (row 1, columns A, B, C, and D)

CRA models have also been used for public health economic modeling of NCDs [30–32]. These are commonly aggregate-level models that use population-attributable fractions to estimate how parameters describing the relationship between a risk factor and disease outcome would change following an intervention. Individuals can be simulated when combined with microsimulation (see section: *Use of microsimulation with individual-level decision tree, comparative risk assessment, and Markov models*). CRA models do not allow for interactions but can simulate the age- and sex-specific effect of an intervention on multiple risk factors and disease processes simultaneously without becoming as complex as the equivalent decision tree. Other population strata can be simulated in the same way, thereby estimating the impact on health inequalities. Furthermore, CRA models can be adapted to include a time component (see section: *Using multistate life tables*). Costs and morbidity associated with each disease process can be used to compare total cost and health outcomes with and without an intervention. Examples including costs are the WHO Comparative Risk Assessment project [30], a French model simulating changes to fruit and vegetable consumption [31], and a US model by Trogdon et al. estimating the potential return on investment of workplace obesity interventions [32]. Trogdon et al.’s model estimates some outcomes and costs outside of the health sector by capturing costs from the employer perspective. This approach of estimating wider societal costs and consequences directly from the health outcome (such as lost days at work as a result of sickness) can be applied to all model structures discussed in this paper where data on the societal outcomes and how they relate to the health outcome are available.

#### Markov models without interaction (row 2, columns A, B, C, and D)

In comparison to decision trees and CRA models, Markov models are much more commonly used in public health economic modeling of NCDs, and are able to simulate more complex scenarios [28]. For example, a Markov model may be used to model the cost-effectiveness of different smoking cessation methods incorporating multiple disease outcomes, the probability of relapse, and outcomes over different time horizons [33–35]. Markov models simulate how a population or individual moves between predefined health or disease states at a specific time interval (for example, annually). This incorporates a time component and allows for modeled populations to remain in a health state from one time interval to the next, and to loop back from a diseased state to a healthy state (recur), all based on a given transition probability. The model can then be run for either a predefined number of cycles or, if using a population cohort, until the entire population have reached a certain age or died. In this way, long-term effects of interventions on disease outcomes can be estimated. When computing cost-effectiveness from a Markov model, each health state is assigned a measure of disease morbidity and cost. As the model runs through cycles, the costs and morbidity (or utility) can then be summed for the numbers of individuals in each state for each time cycle.

Markov models can be relatively straightforward to develop and to represent graphically, thereby making them transparent to peer-reviewers and decisionmakers. Furthermore, transitional tunnel states can be added to increase complexity and make the model more realistic. For example, in order to more accurately capture the increased costs and morbidity associated with the first year of having a heart attack compared to subsequent years, all individuals who have a heart attack may spend one cycle in a tunnel state with associated high morbidity and cost, before having a 100 % probability of leaving that state. Markov models have been commonly used in a range of different public health economic analyses of NCDs in different settings and countries, for example the Dutch Rijksinstituut voor Volksgezondheid en Milieu (RIVM) Chronic Disease Model (CDM) [36–39], the Benefits of Smoking Cessation on Outcomes (BENESCO) model [33–35], the Australian Quit Benefits Model [40, 41], the US Centers for Disease Control and Prevention (CDC) Measurement of the Value of Exercise (MOVE) model [42], the Australian Coronary Heart Disease Prevention Model [43], the US Coronary Heart Disease (CHD) Policy Model [44, 45], and bespoke models for analyzing public health interventions internationally [46], in the Netherlands [47], Switzerland [48], Finland [49, 50], Germany [51], US [52, 53], UK [54, 55], and in Australia [56, 57].

Markov models have some important assumptions. Firstly, population-level models have no memory (the Markovian assumption) meaning that the morbidity, cost, and transition probabilities associated with a given health state are the same irrespective of previous health states or how long an individual has been in a health state. This can, in part, be dealt with by using tunnel states, as used by the Australian Quit Benefits Model [40], or by using microsimulation (see section: *Use of microsimulation with individual-level decision tree, comparative risk assessment, and Markov models*). Secondly, individuals can also only exist in one state at a time. This means that to add a new disease to a model that can coexist with the previously modeled diseases, the number of health states included in the model must each time be exponentially increased (as new health states are required for each disease combination). Further complexity is introduced if trying to model a heterogeneous population where population subgroups have different transition probabilities. This can be addressed by using weighted average costs, disabilities, and transition probabilities at the aggregate level. Alternatively, multiple cohorts, each with different characteristics, can be run through the model with cohort-specific transition parameters to give results by population subgroup, which can also be useful when estimating effects on inequalities; however, this adds model complexity and run time. An example of using multiple Markov models to model a heterogeneous population with multiple disease states is the US CDC diabetes prevention model, discussed in more detail in section: *Markov chain models and individual-level Markov models with interaction* [58, 59].

### Rows 3 and 4 - interaction allowed

#### System dynamics models (rows 3 and 4, column A)

System dynamics models allow for populations to interact both with each other and with their environment. The probabilities of events occurring in the model (the system) change through feedback as the model runs, governed by algebraic or differential equations [60]. Such a model can be made increasingly complex as increasing numbers of factors influencing the system are added (requiring increasing amounts of data). This makes system dynamics models better able to simulate interactions within complex non-health sector systems, and to estimate effects of multicomponent interventions than previously discussed model structures. Costs can be applied to either the disease state, or to all factors within the model, and then cost and health outcomes with and without the intervention can be compared. System dynamics models can usually be represented graphically, facilitating communication of the model with stakeholders. Such models are well-established for communicable diseases [61, 62] and are increasingly being applied to NCD risk factors, such as Macmillan et al. who used a system dynamics model to explore the potential effect of different transport policies on bicycle commuting in Auckland, New Zealand [63]. The authors not only estimated health outcomes, but also the effect on air pollution, carbon emissions, and fuel costs over a period of 40 years. In this way, long-term health and economic impacts were estimated, and some non-health outcomes were quantified. The authors monetized the model’s outcomes and a cost-benefit analysis was used to compare different policies. Through monetizing non-health outcomes and assigning utilities to health outcomes it would be possible to perform a cost-effectiveness analysis using the same approach. A potential limitation of system dynamics models is that the dynamic element of the model (the rate of change of parameters with time) is deterministic, although parametric uncertainty can be modeled.

#### Markov chain models and individual-level Markov models with interaction (rows 3 and 4, columns B and C)

In discrete or continuous time Markov chain models, state transition probabilities can depend on (interact with) the proportion of different populations in different disease states, and on the time that has elapsed in the model. These interactions are the key difference between Markov chain models and those discussed in section: *Markov models without interaction*, and provide the model with some degree of memory, in part overcoming the Markovian assumption. However, when simulating cohorts using time-dependent transition factors, it is not possible to completely overcome this assumption because the model cannot know how long different proportions of the population in any given health states have been in that state. By contrast, individual-level Markov models simulate individuals separately (also called microsimulation, see relevant section on microsimulation later in article), making it possible to ”know” how long an individual has been in each state and to alter transition probabilities as a function of time in a given state. The simulation may or may not retain “memory” of which states an individual has been in previously. Markov chain and individual-level Markov model structures with interaction are better able to cope with system complexity than CRA and decision tree model structures, however they are unable to explicitly model non-health sector system interactions.

Examples of discrete time aggregate-level Markov models allowing for interaction are a version of the RIVM CDM (also cited in *Markov models without interaction (row 2, columns A, B, C, and D)* section) which includes disease incidence parameters that depend on time from smoking cessation [64], and the US CDC diabetes prevention model, described in detail in a technical report by Hoerger et al. available from Herman et al. as an online supplement [59]. In this cohort model, transition probabilities are dependent on time since diagnosis of diabetes, as well as on levels of glycemia and hypertension. Furthermore, the model simulates multiple disease processes simultaneously by allowing the cohort to coexist in five different disease pathways which are linked to the overall Markov model by bridge models (see online supplement from Herman et al. for full description of the model) [59, 65]. Bridge models allow the overall Markov model to collect accumulated data on the number of events that have occurred, and keep track of the proportion of the cohort remaining in each disease state in any given year and the proportion who have left either through death or remission. Finally, Herman et al. account for a heterogeneous population by simulating 560 different cohorts, each with individual state transition probabilities dependent on age, sex, race, hypertension, cholesterol, and smoking status.

#### Discrete event simulation (rows 3 and 4, column D)

Discrete event simulation (DES) is an extremely flexible modeling structure that simulates a system changing over time with a sequence of discrete individual events [66]. Rather than simulating populations or individuals through states for a fixed length of time, multiple future events are in competition and the model jumps to whichever event occurs next based on predefined probabilities. The occurrence of an event can directly lead to a series of contemporaneous events, as well as affect the probability of future events. The probability of a given event occurring can also vary with time and be affected by interactions between individuals, populations, and their environment at each event. A set of system rules and probabilities govern the behavior of the population or individuals in a DES model, and these can be changed based on the intervention being modeled. As each event occurs, costs and utilities based on the event, consequence, and time to event are estimated. However, due to the large number of variables possible in DES and the need to simulate many individuals, models can be computationally slow to run (particularly when estimating uncertainty) and require large quantities of data for each disease outcome. Within public health economic modeling of NCDs, this approach has been commonly adopted for evaluating screening programs. [67] Along with other model structures that can simulate interactions between the population being modeled and the environment, DES is well-equipped for addressing interactions within complex systems.

### Row 5 – interaction between heterogeneous entities/special aspects important

#### Agent-based simulation (row 5, column D)

Agent-based simulation (ABS) has many similarities to DES in that it allows for the probability of events occurring within the system being modeled to change as a consequence of interactions between individuals (agents), between agents and the environment, and with time. ABS is therefore also able to deal with the challenges of multicomponent interventions and interactions within complex non-health sector systems. The difference, however, is that ABS models apply rules to agents or groups of agents, and responses depend on individual agent characteristics which can change either over time or following interactions with other agents or the environment. This is compared to system-based rules found in DES [22, 68]. A heterogeneous population of agents is therefore able to ”learn” over time and affect the system, which, as Squires discusses, allows for more accurate representation of spatial effects, such as social networks [22]. However, ABS is again more complex than DES and can require considerably more data to represent heterogeneous population groups. Costs and morbidity can be applied to each event and disease state in order to derive estimates of cost-effectiveness of interventions that affect the behavior of agents (other societal costs and outcomes could also be estimated in a similar fashion). Population-level results emerge from the aggregate of all agent-level results.

An example of ABS being used in public health economic modeling of NCDs is the Archimedes Model [69, 70]. This model was designed to simulate a wide range of interventions modeling a whole suite of clinical outcomes through changes to physiological risk factors. It is therefore a good example of simulating multicomponent interventions. It allows interactions between variables both within and between individuals, and with the system as a whole. As such, it relies on a large amount of processing power and data.

## Using multistate life tables and microsimulation to increase model flexibility

### Use of multistate life tables with decision tree, comparative risk assessment, and Markov models with no interaction (rows 1 and 2, columns A, B, C and D)

For the purposes of this review, multistate life tables are defined as life tables that model an individual’s, or proportion of a population’s, probability of developing a given disease at different ages and subsequent case fatality rates once the disease is acquired. These can simulate multiple diseases simultaneously and can be used to add a temporal component to decision tree or CRA models. For each intervention scenario being analyzed, the decision tree or CRA model can be used to generate population impact fractions to alter the multistate life table disease incidence, case fatality, and overall mortality rates. Rerunning the multistate life-table model then allows scenarios to be compared over the number of years in the life table allowing for long-term health effects and economic impacts to be estimated.

In a similar way, multistate life tables can be used with population or individual Markov models to simulate multiple diseases without the need for large increases in the number of disease states. To do this, proportions of the population can coexist in more than one disease state in the multistate life table and for each disease a Markov model can simulate the probability of moving between diseased and not diseased states (thereby performing a similar function to bridge models, as discussed in section: *in Markov chain models and individual-level Markov models with interaction*). These properties usually assume that diseases are independent of one another (for example, the probability of developing ischemic heart disease does not change with a concurrent diagnosis of cancer). Published examples include the Australian Assessing Cost Effectiveness in Prevention (ACE Prevention) program of research (which estimates the effects of various interventions over the lifetime of the Australian population) [71–76], the WHO PopMod model (which estimates effects over a 100-year time period) [77, 78], and a New Zealand model estimating the effects of increasing tobacco taxation (which estimates effects over the lifetime of the New Zealand population) [79].

### Use of microsimulation with individual-level decision tree, comparative risk assessment, and Markov models (rows 1 and 2, columns C and D; and rows 3 and 4, column C)

In order to overcome the complexity of modeling multiple diseases and heterogeneous populations in decision tree, CRA, and Markov model structures, an alternative approach is to use individual patient simulation models (microsimulation). These allow for a population of heterogeneous individuals to move through the model based on probabilities appropriate to their characteristics (such as demographic factors or physiological characteristics). The model is run at the individual level with all members, or randomly selected members of a predefined population, being simulated until either a prespecified outcome occurs or a certain length of time has elapsed (e.g., death or reaching age 100). Once completed, the individual results can be aggregated to calculate a single population-level result or for percentile (or other) variations in results across individuals to be reported (thereby also allowing reporting of inequalities). Microsimulation is also particularly useful when estimating population means of skewed effects (such as the growth rates of different cancers when a few may be very quick-growing and have different subsequent events compared to the majority being slow-growing), and is very flexible at modeling interactions.

Model parameters can be changed for different scenario analyses in the same way as with multistate life tables see section: *Use of multistate life tables with decision tree, comparative risk assessment, and Markov models with no interaction* by using decision tree, CRA, or Markov models. However, in microsimulation this is at the individual level and parameters are specific to the individual’s characteristics (such as their age and gender). Drawbacks of individual-level simulation are that they are computationally intense although modern computers and software are increasingly able to cope with the many thousands of iterations often computed. Examples of patient-level simulation Markov models within public health economic modeling of NCDs include the UK Health Forum model [80], the World Health Organisation and Organisation for Economic Co-operation and Development (OECD) Chronic Disease Prevention (CDP) model [81, 82], and the NICE Obesity Health Economic Model [83], as well as examples from Australia [84], Korea [85], and Sweden [86, 87].

## Conclusions

In the context of an ever-increasing global burden of preventable NCDs, decisions need to be made as to how to approach their prevention and management. Modeling the cost-effectiveness of NCD-related public health interventions is an expanding academic field that is starting to embrace more sophisticated modeling structures (see Table 2 for a summary of model structures). Public health economic modeling of NCDs has thus far primarily used Markov models, with or without the use of life tables and microsimulation, to investigate the health impacts of a given intervention and its cost-effectiveness. However, there is scope for more complex systems to be modeled with a wider range of possible outcomes by using model structures such as DES and ABS. Balancing transparency and parsimony with complexity when developing such models is crucial for model results to be readily interpreted and used by decisionmakers [24, 88–90].

We identify many NCD public health economic models that, to a greater or lesser extent, address the specific challenges of epidemiological, public health, and economic modeling when compared to health technology appraisals; namely assessing long-term health and economic impacts, quantifying societal costs and consequences, identifying the potential impact on inequalities, simulating multicomponent interventions, and simulating interactions within complex non-health sector systems.

The solutions to these challenges presented in this review are either specific to a given model structure, or can be applied across all structures. Long-term health and economic impacts are impossible to estimate using cross-sectional model structures such as CRA and decision tree models structures. However, combining these with a multistate life table can add a longitudinal component to the model thereby enabling long-term outcomes to be simulated [74, 78].

Wider societal costs and consequences (e.g., air pollution or effects on employment) can be estimated using any model structure. The challenge is less a problem of choosing the appropriate epidemiological modeling structure, but more an issue of identifying robust cost and non-health data with which to parameterize the model; although, some model structures are more adaptable than others for estimating multiple non-health outcomes. For example, if data are available it may be possible within any model structure to estimate some of the effects on productivity costs of an intervention directly from a model’s health outcomes by inferring what may happen to work absenteeism as a consequence of ill health [29]. It is less easy to do this for non-health related outcomes such as effects on air pollution as a result of changes to traffic policy and numbers of cyclists. Macmillan et al. do this using a system dynamics model and relate air pollution (and its cost) to part of the causal pathway (number of vehicles on the road) between the intervention and health outcome [63]. For outcomes that cannot be directly estimated from a step on a model’s causal pathway or result from interactions with other model outcomes (for example, if increased air pollution affected weather patterns which in turn affected cycling habits) then model structures allowing for interactions are more appropriate.

The potential impact of a simulated intervention on inequalities can also be estimated using any epidemiological modeling structure by simulating population groups of interest separately if using a cohort model (for example, Blakely et al. estimated the effects of a tobacco tax by ethnic group in New Zealand using a multistate lifetable model [79]) or aggregating results from the two or more population groups of interest when using microsimulation (such as that used by Feldman et al. when estimating the effects of lifestyle interventions on different risk groups for metabolic syndrome [87]). Estimating wider societal costs and consequences is dependent on available data.

The challenge of multicomponent interventions can be addressed both using epidemiological model structures that do and do not allow for interaction. Without interaction, a model assumes that each component of a multicomponent intervention acts independently on a disease outcome or risk factor. If interaction between multiple interventions is necessary to simulate, then model structures that allow interactions are required. Both for multicomponent interventions and for simulating interactions within complex non-health sector systems, microsimulation models (such as ABS and DES) offer more flexibility than population-level models.

However, it is important to note that when dealing with any of these challenges, a model is only as good as the data used to parameterize it and adding more complexity may only serve to make the model more uncertain and more difficult to communicate. As Whitty discusses, a model that is simple, timely, and lays bare its problems is far more useful to a policymaker than one that is more detailed, more complicated, possibly more accurate, but less interpretable and arrives after the policy decision is made [90].

No one model identified in this review addresses all the challenges of modeling economic evaluations of public health interventions for NCDs, and the choice of which epidemiological model structure to adopt will depend on what is being modeled: the interventions being evaluated, the outputs required, and the needs of the decisionmaker. We therefore provide a revised version of Brennan et al.’s taxonomy of model structures for the economic evaluation of health technologies to act as a guide to modelers in the field of public health economic modeling [24].
